# Acute traumatic brain injury in frail patients: the next pandemic

**DOI:** 10.1097/MCC.0000000000000915

**Published:** 2022-01-25

**Authors:** Marta Baggiani, Angelo Guglielmi, Giuseppe Citerio

**Affiliations:** a University of Piemonte Orientale, Novara; b University of Pavia, Pavia; c School of Medicine and Surgery, University of Milano-Bicocca, Milano; d Neurointensive Care Unit, San Gerardo Hospital, ASST-Monza, Monza, Italy

**Keywords:** brain injury, elderly, falls, frailty

## Abstract

To highlight recent findings on the evaluation and impact of frailty in the management of patients with traumatic brain injury (TBI).

**Recent findings:**

Frailty is not a direct natural consequence of aging. Rather, it commonly results from the intersection of age-related decline with chronic diseases and conditions. It is associated with adverse outcomes such as institutionalization, falls, and worsening health status. Growing evidence suggests that frailty should be a key consideration both in care planning and in adverse outcome prevention. The prevalence of elderly patients with TBI is increasing, and low-energy trauma (i.e., ground or low-level falls, which are typical in frail patients) is the major cause. Establishing the real incidence of frailty in TBI requires further studies. Failure to detect frailty potentially exposes patients to interventions that may not benefit them, and may even harm them. Moreover, considering patients as ‘nonfrail’ purely on the basis of their age is unacceptable. The future challenge is to shift to a new clinical paradigm characterized by more appropriate, goal-directed care of frail patients.

**Summary:**

The current review highlights the crucial importance of frailty evaluation in TBI, also given the changing epidemiology of this condition. To ensure adequate assessment, prevention and management, both in and outside hospital, there is an urgent need for a valid screening tool and a specific frailty-based and comorbidity-based clinical approach.

## INTRODUCTION

Frailty is characterized by a decline in functioning across multiple physiological systems, accompanied by increased vulnerability to stressors, and it is becoming increasingly common as a result of the global aging of the population.

Although the global burden and prevalence of frailty are not well known, some general patterns have emerged: it is more frequent in women than men, increases with aging (even though it is present in all age groups), and is more common in lower socio-economic groups and ethnic minorities [1,2]. Evidence is growing around the impact of frailty in the ICU [3–6] and after severe brain damage [7^▪^,^8^,9^▪^,^10^,^11^,12^▪^]. Frailty is associated with higher mortality in older patients and is related to a broad spectrum of ‘geriatric conditions’ such as dementia, cognitive decline, disability, falls, fractures, loneliness, worsening mobility, lower quality of life, hospitalization, and dependence on home nursing [13].

The aim of this manuscript is to review the existing instruments for describing frailty, to evaluate the role of frailty after a traumatic event, and to suggest potential interventions for blunting its impact in the ICU and after traumatic brain injury (TBI).

### How to capture the frailty phenotype

Frailty is, by definition, a multidimensional condition. Not surprisingly, numerous instruments have been developed to measure it, including objective assessments, self-reports, performance assessments, or combinations of the three (Table 1). Although numerous, they generally fall into two general models, as also suggested by recent reviews published in *The Lancet*[13,14].

**Table 1:** Recent updates of frailty measurements tools

Article	Type and number of patients	Type of study	Mean age	Assessment/screening tool	Findings
Spiers GF, Kunonga TP, Hall A *et al.*[7^▪^] 2021	General population More than 2.8 million patients	Systematic review	70.4% of studies: 51–59.9 years old 24.1% of studies: 41–50 years old 4.7% of studies: 31–40 years old 0.8% of studies: 18–30 years old	41 measures of frailty including: Cumulative deficit frailty indices Phenotype measures FRAIL Scale SPPB CFS LFI JHFI OFFS CHSFS	Frailty measures have limited evidence of predictive validity in younger population not only in elderly
Pecheva M, Phillips M, Hull P *et al.*[8] 2020	Trauma 819	Retrospective cohort review	76.3 years old	mFI	Increased frailty is associated with: increased mortality (both at discharge and at 1 year from discharge) and higher rate of serious complications (unplanned intubation, infection, progressive renal failure and ventilator dependency) low frailty patients were more likely to be admitted to ICU than high frailty patients (49.8 vs. 32.7%) high frailty patients were less likely to be discharged to their own home compared to low frailty patients (29.1 vs. 40.5%)
Zhao F, Tang B, Hu C *et al.*[10] 2020	Trauma 50348	Systematic review	>50 years old	CFS GFI VMS mFI TSFI Hip-MFS MFC	Frailty in elderly patients impacts on adverse posttraumatic outcomes
Rickard F, Ibitoye S, Deakin H *et al.*[9^▪^] 2021	Trauma 300	Prospective observational	82 years old	CFS	In traumatized older patients, frailty is an independent predictor of 30-day mortality, inpatient delirium and increased level of care at discharge
Tracy BM, Carlin MN, Tyson JW *et al.*[12^▪^] 2020	TBI 2352	Retrospective observational	57 years old	mFI-11^a^	Frailty in TBI patients is associated with: greater rates of discharge to unfavorable locations (facilities) increased odds of acute kidney injury unplanned events among patients with TBI
Rahul A. Sastry, Nathan Pertsch, Oliver Tang *et al.*[27] 2020	TBI 1647	Retrospective observational	69.35 years old	mFI-5^b^	In a cohort of patients undergoing craniotomy for chronic subdural hematoma, preoperative evaluation of frailty is correlated with: increased number of major postoperative complications discharge to destination other than home 30-day mortality
Pierce KE, Naessig S, Kummer N *et al.*[25] 2021	Spine surgery 61 356	Retrospective observational	57 years old	mFI-5^b^ vs. mFI-11^a^	Frailty is correlated with increased morbidity and mortality The preoperative calculation of frailty with mFI-5, which is a shorter and readily accessible tool, can predict postop complications similarly to mFI-11
Pazniokas J, Gandhi C, Theriault B *et al.*[26] 2021	Neurosurgery 162 375	Systematic review	4/5 studies >65 years old 1/5 study >50 years old	mFI mFI-5^b^ CFS Novel Preop Frailty Scale FRAIL Scale Spinal Frailty Index Metastatic Spinal Tumor-FI CD-FI ASD-FI	Increased frailty is associated with worse patients’ outcome in neurosurgery
Utino Taniguchi L, Ibrahim Q, Azevedo LCP de *et al.*[21^▪^] 2020	Critical Ill Patients 421	Posthoc analysis of multicenter prospective cohort	67.15 ± 9.87 years old	CFS mFI-11^a^	Low concordance between CFS and mFI Both CFS and mFI-11 have predictive validity for hospital mortality

ASD-FI, Adult Spinal Deformity Frailty Index; CDFI, Cervical Deformity Frailty Index; CFS, Clinical Frailty Scale; CHSFS, Cardiovascular Health Study Frailty Scale; GFI, Groningen Frailty Indicator; Hip-MFS, Hip-Multidimensional Frailty Score; JHFI John Hopkins Frailty Indicator; Liver Frailty Index LFI; MFC, Modified Fried Criteria; mFI, modified Frailty Index; OFFS, Study of Osteoporotic Fracture Frailty Scale; REFS, Reported Edmonton Frail Scale; SPPB, Short Physical Performance Battery; TSFI, Trauma-Specific Frailty Index; VMS, Veiligheids Management System.

a 11-mFI variables: history of diabetes mellitus, history of hypertension requiring medicaments, history of either transient ischemic attack and cerebrovascular accident,functional status 2 (not indipendent), hystory of miocardial infarction, hystory of either peripheral vascular disease or rest pain, hystory of cerebrovascular accidents with neurological deifict, hystory of COPD or pneumonia, hystory of either prior percutaneous coronary intervention or prior cardiac surgery or angina, history of impaired sensorium, history of congestive heart failure.

b 5-mFI variables: history of diabetes mellitus, functional status 2 (not indipendent), history of hypertension requiring medicaments, history of COPD or pneumonia, history of congestive heart failure.

The first model considers frailty to be a biological syndrome, identifiable as a distinct phenotype. The Cardiovascular Health Study Index, developed by Fried *et al.* (often referred to as the Fried Frailty Phenotype [15]), is the most widely cited example of this model. It quantifies deficits in five domains with frailty being defined as the presence of deficits in at least three of them.

The second model, or cumulative deficit approach, defines frailty by enumerating health abnormalities; in this case, less attention is paid to the specific nature or severity of each problem. Based on this concept, Rockwood and colleagues developed a cumulative deficit frailty instrument, or frailty index [16,17].

The original frailty index includes clinical features, functional characteristics and laboratory measures that are known to be associated with the development of adverse outcomes. Importantly, it is a multidimensional construct, not just a measure of multimorbidity. Deficits in its items are counted, and a frailty index score is then obtained by counting the number of deficits present out of the number measured.

Searle *et al.*[18], too, maintain that the principle for measuring frailty should be to count health deficits, the rationale being that the more deficits a person has, the more likely that person is to be frail. Although the items included in a frailty index (symptoms, signs, diseases, disabilities, laboratory, radiographic, or electrocardiographic abnormalities) may vary in number, it is important to include enough to ensure precise estimates: indeed, ‘estimates are unstable when the number of deficits is small – about 10 or less’. Notably, in the various cohorts in which the frailty index approach has been used, the variables considered were population relevant, that is, selected to best reflect the study population's posited deficits. As Searle and other experts in the field agree, it does not matter if one clinical condition carries a different weight in terms of outcome prediction as compared with another condition. What does matter when constructing a frailty index is to ensure that the variables included meet the following five requisites [18]:

(1) They must be deficits associated with health status.
(2) Their prevalence must generally increase with age.
(3) They must not saturate too early.
(4) As a group, they must cover a range of systems.
(5) If the frailty index is to be used serially on the same people, its items need to be the same from one iteration to the next.

The literature contains evidence, reviewed by Spiers *et al*. [19^▪^], of predictive validity for cumulative deficit frailty index measures in general populations, diagnostic-specific populations (HIV, systemic lupus erythematosus, metabolic syndrome, survivors of myocardial infarction, childhood survivors of cancer, chronic kidney, or end-stage renal disease), and surgical populations (hematopoietic cell transplant or other nonspecified surgery). 

**Box 1:**
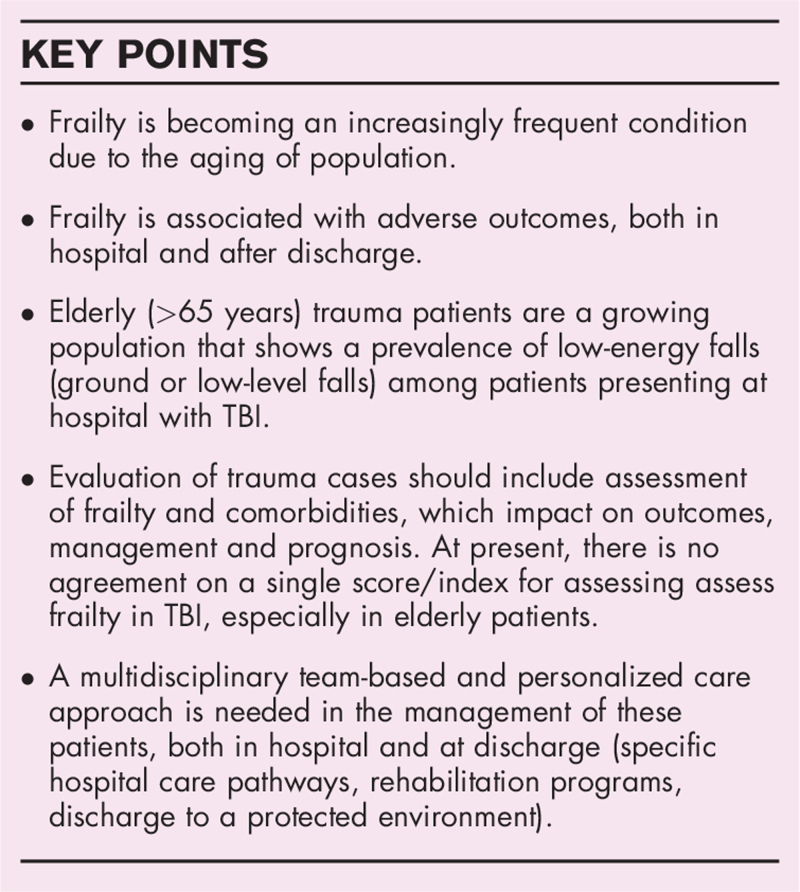
no caption available

### Frailty in trauma patients

Frail older patients who experience major trauma show direct increased mortality, confirmed both at discharge and 1 year after the acute event [20^▪^,21^▪^].

Moreover, frailty is associated with a higher rate of serious complications and consequently with the need for discharge to rehabilitation facilities or other special care settings. Even frail patients who had not required ICU admission and were discharged alive were found to require a lengthy period of in-patient rehabilitation [20^▪^].

A recent systematic review and meta-analysis including a large cohort of elderly trauma patients (more than 50 000) explored the impact of frailty on outcomes. Frailty was identified as a significant predictor of mortality, more so when evaluated in hospital and at 30 days [odds ratio (OR), 4.05; 95% confidence interval (CI), 2.02–8.11; *I* = 0%] than when evaluated after 30 days (OR, 2.41; 95% CI, 1.17–4.95; *I* = 88.1%), and it predicted all postoperative complications (OR, 2.23; 95% CI, 1.34–3.73; *I* = 78.2%), especially Clavien Dindo IV complications (OR, 4.16; 95% CI, 1.70–10.17; *I* = 0%). Furthermore, frailty was correlated with adverse discharge disposition (OR, 1.80; 95% CI, 1.15–2.84; *I* = 78.6%) and with hospital readmission (OR, 2.16; 95% CI, 1.19–3.91; *I* = 21.5%). The authors also performed a subgroup analysis in which prospective studies were shown to be superior to retrospective ones in correlating frailty with postoperative complications (OR, 3.06; 95% CI, 1.43–6.56) [22^▪^].

The optimum frailty assessment in geriatric trauma patients is still debated. A systematic review by Cubitt *et al.*[23] concluded that even though early assessment would be useful to prevent adverse outcomes, no evidence supports the adoption of any particular index or score.

In hospitalized frail trauma patients, unplanned ICU admission (UIA) has been shown to highly correlate with age and greater frailty [7^▪^,^24^^▪▪^].

In the United States, UIAs have already been used as a means of assessing quality of care in trauma patients [25]. Mulvey *et al.*[7^▪^] were the first to examine their effect in a geriatric trauma population: in their retrospective analysis, 2923 geriatric trauma patients (>65 years) were tracked to evaluate UIA-related predictive risk factors. UIA was correlated with higher morbidity and a more than two-fold increase in mortality. However, only age and Injury Severity Score (ISS) were statistically predictive of UIA.

Nevertheless, age did not appear to be a clinically significant variable, with a difference of only 3 years found between UIA and non-UIA patients. Similarly, the median ISS value (=10), despite its statistical significance, did not represent severe trauma.

According to Mulvey *et al.*[7^▪^], geriatric trauma patients are also more prone to in-hospital complications such as unplanned intubation, infections, deep vein thrombosis, sepsis and acute respiratory distress syndrome, and patients who were ‘bounced back’ to ICU (69.5% of the overall number of UIA cases) had worse outcomes.

The fact that cardiac and respiratory complications were the most common reasons for these ‘bounce backs’ suggests that frail geriatric patients discharged from ICUs may warrant a higher level of care [7^▪^].

### Frailty and traumatic brain injury

**FIGURE 1:**
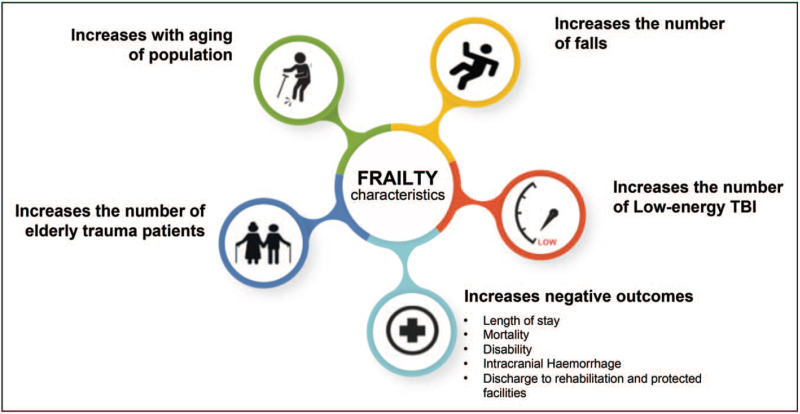
Features and progressive evolution of frailty in traumatic brain injury.

The last decade has brought a change in the pattern of traumatic injury (a prevalence of low-energy accidents) and an increased frequency of TBI in elderly people (>60 years) (Fig. 1) [8].

In the recent prospective, multicenter, longitudinal cohort study CENTER TBI (Collaborative European NeuroTrauma Effectiveness Research), the overall median age of TBI patients was 50 years (interquartile range 30–66) with 28% (1254 patients) older than 65 years [26].

Frailty is related to a broad range of outcomes other than mortality, and these include an increased risk of falls [13].

In a retrospective review, postfall hospital readmission of octogenarian patients correlated directly with mortality at 6 months. Anticoagulation drugs showed no effect on mortality, corroborating frailty as the stronger predictor of readmission and mortality [9^▪^].

A recent study of 21 681 TBI patients, conducted using an efficient registry methodology enabling a real-world approach, showed that at least 40% had ground or low-level (low-energy transfer) accidents. Compared with patients injured as a result of high-energy transfer accidents, this group (the LE cohort) was generally older (>65 years), predominantly female, and more frequently had preexisting conditions associated with the use of anticoagulant and antiplatelet drugs; these findings confirm the increasing proportion of elderly trauma patients in advanced trauma centers. The LE patients normally presented at the emergency department with mild TBI, were less likely to present at hospital intubated, and had better vital signs and normal pupils. Overall mortality was similar following low-energy and high-energy accidents, but four times greater in the LE patients admitted to the ward. Moreover, patients suffering low-energy falls were half as likely as high-energy TBI patients to receive intensive care or emergency intervention [9^▪^]. Since energy transfer does not seem to be predictive of TBI severity in older people, further studies are needed to evaluate the appropriateness of choosing less intensive care in geriatric trauma patients [27].

Polypharmacy (the use of multiple anticoagulant or antithrombotic drugs) has been shown to be associated with evolving lesions on computed tomography scans, more extensive extra-cranial lesions, hemorrhagic progression, and delayed intracranial hemorrhage, with no correlation with adverse functional and clinical outcomes [28^▪^]. Frail patients are more likely to require hospitalization; they are also more prone to adverse events, and therefore face the prospect of persistent posttraumatic deficits that lower their quality of life. Moreover, the elderly population after TBI have a higher probability of an unfavorable outcome, due to their preinjury health status and age [10].

In recent years, few studies have specifically analyzed the correlation between frailty and TBI, and their association with negative outcomes. In a retrospective review by Tracy *et al.*[24^▪▪^], 2352 patients with TBI (>16 years) were classified according to their frailty calculated using an 11-variable modified frailty index. Frailty was found to increase the odds both of acute kidney injury (OR 2.06, 95% CI 1.07–3.99, *P* = 0.03) and of any unplanned event (OR 1.6, 95% CI 1.1–2.3, *P* = 0.01). Furthermore, in agreement with Hatcher *et al.*[29], frail patients were found to have a greater frequency of adverse discharge dispositions. The latter also found that frail patients admitted for falls, where frail corresponded to a Clinical Frailty Scale (CFS) score more than 5, showed a higher rate of readmission for the same reason.

Conversely, these two studies differed with regard to their data on hospital length of stay (LOS), ICU LOS, and ventilation duration, with Tracy *et al.* finding no differences between frail and nonfrail patients [24^▪▪^,^29^].

The difference probably lies in their use of different methods to evaluate frailty: mFI-11 [24^▪▪^] vs. CFS [29]; other authors have subsequently shown these tools to be poorly correlated [30^▪^].

TBI is a complex clinical condition whose impact on some domains of frailty becomes crucial. In this regard, it is necessary to consider not only the biological lesion, but also the patient's pre and posttraumatic psychological conditions.

A recent observational study evaluated elderly patients (>60 years) with mild TBI. They were followed up at three time points: 2 weeks, 1 year, and 3 years. At 2 weeks, posttraumatic complaints and emotional distress were evaluated, while at 1 and 3 years, frailty was calculated, using the Groening Frailty Indicator and the Glasgow Outcome Scale Extended [11]. The frail patients had worse long-term outcomes (three times more likely to have unfavorable outcomes); and compared with age, early complaints were a stronger predictor of unfavorable outcome. On the basis of these findings, mild TBI patients might benefit from more specific follow-up and therapy/rehabilitation to improve their outcomes [11,31^▪▪^].

Another TBI-related clinical condition that has increased in frequency because of the expanding elderly population is acute subdural hematoma (aSAH) [27].

Evans *et al.*[32] in their systematic review tried to identify factors that might predict postoperative outcome of aSAH, with a view to identifying patients that would benefit from surgical management. Given the lack of literature, the review, as the authors expected, failed to provide high-quality evidence that may aid in physiological and clinical prognostic evaluation of this population.

A recent longitudinal, prospective, observational study was performed to evaluate data on patients presenting within 24 h of a TBI at 65 centers in Europe over a 2-year period [33^▪▪^]. A cumulative deficit approach was used to create a novel frailty index from data available in the CENTER-TBI database. The CENTER-TBI frailty index was standardized (range 0–1), with high scores indicating higher levels of frailty. The overall median CENTER-TBI frailty index score calculated from 2993 participants was 0.07 (Q1–Q3 = 0.03–0.15); older patients had a higher score: 0.17 (Q1–Q3 = 0.08–0.27). In this TBI cohort, frailty was significantly associated with the probability of death or severe disability (cumulative OR = 1.03, 95%CI 1.02–1.04, *P* < 0.0001). This association was even stronger in patients admitted to hospital wards (1.04, 95%CI 1.03–1.06, *P* < 0.0001) compared with more severe cases admitted to the ICU (1.02, 95%CI 1.01–1.03 *P* < 0.0001). Therefore, the impact of frailty was ‘diluted’ if the TBI was severe. Data from an external validation cohort (*n* = 1667) supported the robustness and reliability of these findings.

A significantly increased risk of unfavorable outcome was found in participants with a high frailty index score, regardless of age. This study suggests the need to consider frailty both in initial evaluation of TBI patients and in TBI prognostic models [33^▪▪^].

The TBI-related literature has some limitations.

First, there is no characterization of TBI lesions (i.e., subdural vs. epidural), even though this aspect may influence clinical presentation.

Moreover, given the different impact of frailty in mild and moderate TBI compared with severe TBI, it would clearly be worth differentiating TBI on the basis of severity.

Finally, in most studies regarding trauma or brain injury, the reference index used was a frailty index; this approach has some limitations as it evaluates only the presence of a comorbidity and not its severity.

### Surgery

Frailty assessment in emergency surgery settings should not be reserved for the older population, since according to Smart *et al.*, frailty can also have a negative impact in younger (>40 years) adults [34,35].

The 5-mFI, (whose items are congestive heart failure within the 30 days prior to surgery, presence of insulin-dependent or noninsulin-dependent diabetes mellitus, a history of chronic obstructive pulmonary disease or pneumonia, partially dependent or totally dependent functional health status at the time of surgery, the presence of hypertension requiring medication), has been validated for many surgical specialties as a more streamlined and user-friendly tool (vs. mFI-11) that can help in preoperative risk stratification by predicting 30-day risk of surgical complications, readmission, nonhome discharge, and mortality [12^▪^,36^▪^].

In a multicenter prospective cohort study, the CFS was used to evaluate 2279 patients of any age; worsening of frailty was found to predict mortality (OR increased by 80% for 90 days mortality), and also poorer outcomes, in all emergency surgical admissions [37^▪^].

It would be useful during preoperative counseling to explain to patients and families the potential additional risks of clinical management procedures and have them participate in the surgical decision-making process; this applies across different surgical specialties [12^▪^,36^▪^].

Flaatten and Beil suggest using the CFS as a simple tool for prediction of outcomes in all fields of anesthesiology, maybe in addition to the well known ASA score in the elderly [38^▪▪^].

## CONCLUSION

Frailty can be considered as the unmeasured heterogeneity between age-matched patients that is strictly linked to mortality risk.

It is a dynamic, multidimensional, often age-related condition that becomes evident when physiological decline reaches a cumulative critical mass [13].

Future challenges related to brain injury and frailty, listed in Table 2, are multifaceted. The heterogeneity of this condition (in terms of presentation, prognosis, and response to treatment) necessarily requires personalized strategies [13,14,31^▪▪^].

**Table 2:** Future challenges in traumatic brain injury frail population

Future challenges:
Identify an optimal tool to evaluate TBI both in ICU and in other departments (neurosurgery, ED, neurology)
Identify biomarkers of frailty to facilitate treatment strategies and monitoring
Identify optimal strategy to manage frailty
Multidisciplinary team-based care, involving geriatric, emergency, and ICU physicians
Discharge to a protected environment
TBI consensus management guidelines to improve elderly long and short-term outcomes

ED, emergency department; TBI, traumatic brain injury.

**FIGURE 2:**
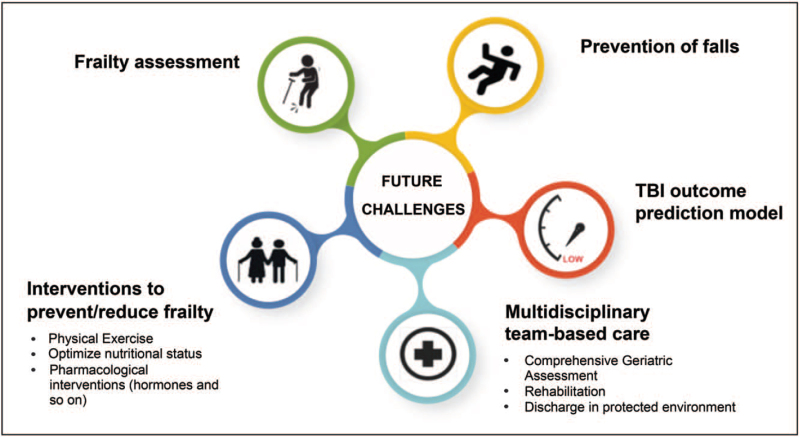
Future challenges in each step of evolution of frailty.

Interventions are possible at different levels: identification, prevention, and treatment (Fig. 2).

Our ability to identify frail people is currently entrusted to existing assessment tools, but there is a clear need to define a proper setting-related gold standard.

Intervening at the level of prevention to reduce prevalence or severity of frailty may benefit patients greatly.

Interventions on modifiable factors for frailty prevention vary and may include physical exercise, nutrition or pharmacological interventions, or comprehensive geriatric assessment [13,14,38^▪▪^,^39^].

Recent publications, focusing particularly on geriatric trauma patients, have also highlighted the importance of providing an additional level of care for frail patients discharged from ICUs, in order not only to reduce readmissions and complications, but also to improve functional outcomes [7^▪^].

Cognitive telerehabilitation performed in the predischarge phase, with the aim of ensuring a higher level of adherence to the home tele-treatment and potentially better outcomes, has been shown to be a promising new cognitive training tool in TBI [40]. Interventions should be combined in a multidisciplinary treatment to obtain better outcomes in frail patients. A recent trial targeting individuals with frailty and sarcopenia evaluated the effectiveness of this combined approach [41].

A future goal would be to improve healthcare systems through the development of targeted frailty care programs. It may be useful, for example, to have the option of discharging frail patients to nurse-assisted healthcare settings (or other facilities), focusing on a personalized model of rehabilitation that takes into account age, frailty, and comorbidities to achieve better long-term outcomes.

On the contrary, data on this topic are currently limited [42,43]. The levels of evidence in this field are low, suggesting that it is still in the early stages of development. Observational studies are predominant rather than well constructed randomized controlled intervention trials. More studies are needed before further conclusions on the most favorable rehabilitation program can be drawn. Geriatric-specific guidelines that incorporate rehabilitation and palliation practices appropriate for this patient population are needed, since frailty is a significant risk factor for adverse outcomes in older patients.

## Acknowledgements

*None.*

### Financial support and sponsorship

*None.*

### Conflicts of interest

*There are no conflicts of interest.*

## REFERENCES AND RECOMMENDED READING

Papers of particular interest, published within the annual period of review, have been highlighted as:

▪ of special interest
▪▪ of outstanding interest
